# Mitigation Effects of Bentonite and Yeast Cell Wall Binders on AFB_1_, DON, and OTA Induced Changes in Laying Hen Performance, Egg Quality, and Health

**DOI:** 10.3390/toxins13020156

**Published:** 2021-02-17

**Authors:** Ling Zhao, Yue Feng, Jing-Tao Wei, Meng-Xiang Zhu, Lei Zhang, Jia-Cai Zhang, Niel Alexander Karrow, Yan-Ming Han, Yuan-Yuan Wu, Yu-Ming Guo, Lv-Hui Sun

**Affiliations:** 1 Department of Animal Nutrition and Feed Science, College of Animal Science and Technology, Huazhong Agricultural University, Wuhan 430070, China; zling@mail.hzau.edu.cn (L.Z.); fengyueyue@webmail.hzau.edu.cn (Y.F.); zhumengxiang@163.com (M.-X.Z.); zhanglei6@webmail.hzau.edu.cn (L.Z.); 2 National Center for International Research on Animal Genetics, Breeding and Reproduction (NCIRAGBR), Huazhong Agricultural University, Wuhan 430070, China; 3 Institute of Animal Husbandry and Veterinary Sciences, Hubei Academy of Agricultural Sciences, Wuhan 430064, China; jintao001@163.com; 4 Animal Husbandry and Fisheries Research Center of Guangdong Haid Group Co., Ltd., Guangzhou 511400, China; zhangjc07@haid.com.cn; 5 Department of Animal Biosciences, University of Guelph, Guelph, ON N1G2W1, Canada; nkarrow@uoguelph.ca; 6 Trouw Nutrition, 773811 Amersfoort, The Netherlands; Yanming.Han@trouwnutrition.com (Y.-M.H.); Yuanyuan.Wu@trouwnutrition.com (Y.-Y.W.); 7 State Key Laboratory of Animal Nutrition, College of Animal Science and Technology, China Agricultural University, Beijing 100193, China; guoyum@cau.edu.cn

**Keywords:** mycotoxins, laying hens, performance, binder, remediation

## Abstract

The objective of this study was to evaluate the efficacy of mycotoxin binders in reducing the adverse effects of co-occurring dietary aflatoxin B_1_ (AFB_1_), deoxynivalenol (DON) and ochratoxin A (OTA) on laying hens. Three hundred and sixty 26-week-old Roman laying hens were randomly allocated into four experimental groups with 10 replicates of nine birds each. The four groups received either a basal diet (BD; Control), a BD supplemented with 0.15 mg/kg AFB_1_ + 1.5 mg/kg DON + 0.12 mg/kg OTA (Toxins), a BD + Toxins with Toxo-HP binder (Toxins + HP), or a BD + Toxins with TOXO XL binder (Toxins + XL) for 12 weeks. Compared to the control, dietary supplementation of mycotoxins decreased (*P* < 0.10) total feed intake, total egg weight, and egg-laying rate, but increased feed/egg ratio by 2.5–6.1% and mortality during various experimental periods. These alterations induced by mycotoxins were alleviated by supplementation with both TOXO HP and XL binders (*P* < 0.10). Furthermore, dietary mycotoxins reduced (*P* < 0.05) eggshell strength by 12.3% and caused an accumulation of 249 μg/kg of DON in eggs at week 12, while dietary supplementation with TOXO HP or XL mitigated DON-induced changes on eggshell strength and prevented accumulation of DON in eggs (*P* < 0.05). Moreover, dietary mycotoxins increased relative liver weight, but decreased spleen and proventriculus relative weights by 11.6–22.4% (*P* < 0.05). Mycotoxin exposure also increased alanine aminotransferase activity and reduced immunoglobulin (Ig) A, IgM, and IgG concentrations in serum by 9.2–26.1% (*P* < 0.05). Additionally, mycotoxin exposure induced histopathological damage and reduced villus height, villus height/crypt depth, and crypt depth in duodenum, jejunum and (or) ileum (*P* < 0.05). Notably, most of these histological changes were mitigated by supplementation with both TOXO HP and XL (*P* < 0.05). In conclusion, the present study demonstrated that the mycotoxin binders TOXO HP and XL can help to mitigate the combined effects of AFB_1_, DON, and OTA on laying hen performance, egg quality, and health.

## 1. Introduction

Mycotoxins are secondary metabolites of fungi, which are toxic to both humans and animals, and are mainly produced by five mold genera: *Aspergillus*, *Fusarium*, *Penicillium*, *Alternaria,* and *Claviceps* [1]. Mycotoxins widely contaminate many crops, including corn, wheat, barley, peanuts, millet, peas, and oily feedstuffs, which have been reported to affect approximately 25% of the world’s agricultural commodities every year [2,3]. Ingestion of mycotoxin-contaminated feed can pose serious negative effects on livestock and poultry health and productivity, and thus cause significant economic losses [4].

To date, more than 400 mycotoxins have been identified in approximately 100 fungi strains [5]. Aflatoxin B_1_ (AFB_1_), deoxynivalenol (DON) and ochratoxin A (OTA) are the primary mycotoxins detected in contaminated agricultural products [3,6,7,8]. AFB_1_ is the most toxic mycotoxin, possessing hepatotoxic, mutagenic, carcinogenic, and teratogenic effects on several species of animals [9,10,11]. In contrast, DON can induce anorexia, vomiting, and impair intestinal and immune function in animals in part by inhibiting the synthesis of nucleic acids and protein [12,13]. OTA is mainly accumulated and metabolized in liver and kidney and induces carcinogenic, hepatotoxic, nephrotoxic, and immunotoxic effects [14]. Since these mycotoxins can coexist in animal feed and have combined cytotoxic effects [6,7,15,16], development of mediation strategies that can prevent or counteract the adverse effects of mycotoxin exposure is a high priority.

In the last two decades, many developments have happened in the area of mycotoxin management and the use of mycotoxin binders in feed has been one of the most practical and effective means. Mycotoxin binders, TOXO HP and XL, are innovations developed to manage multiple mycotoxins in poultry feeds and they are mainly constituted by bentonites and inactive yeast cell wall fractions, including β-1,3/1,6-glucans, in different ratios. These compositions enable the binders to control mycotoxins by three modes of action: (1) Mycotoxin binding to reduce bioavailability, (2) intestinal health protection, and (3) immunomodulation [17,18,19,20]. The objective of the current study was to assess the efficacy of TOXO HP and XL to reduce the combined toxicity of AFB_1_, DON, and OTA to laying hens.

## 2. Results

### 2.1. Laying Performance and Egg Quality

Laying performance results are presented in Table 1. Compared to the control, dietary supplementation of mycotoxins significantly reduced (*P* < 0.10) total feed intake by 2.5% between weeks 9–12, total egg weight by 4.7% between weeks 1–12, and egg-laying rate by 5.2–5.6% throughout the experiment, and increased (*P* < 0.10) feed/egg ratio by 3.7–6.1% between weeks 5–8 and 1–12. Interestingly, the addition of both TOXO HP and XL alleviated these negative effects induced by mycotoxins (*P* < 0.10). Notably, the addition of TOXO XL prevented the mycotoxin-induced adverse effects on total egg weight, feed/egg ratio, and egg-laying rate between weeks 5–8 and weeks 1–12 (*P* < 0.10). Mycotoxins also increased the mortality of laying hens compared to the control during weeks 1–4 and 1–12. Notably, supplementation of TOXO XL mitigated the mycotoxin-induced mortality of laying hens during weeks 1–4 and 1–12. Meanwhile, the addition of TOXO HP prevented or mitigated mycotoxin-induced mortality during weeks 1–4 or 1–12, respectively (Table 1).

Egg quality results are presented in Table 2. Compared to the control, dietary supplementation of mycotoxins did not (*P* > 0.05) affect egg weight, albumen height, egg yolk color, and haugh unit throughout the experiment. Compared to the mycotoxins group, the egg yolk color was improved (*P* < 0.05) in Toxins+XL group at week 12. Notably, dietary supplementation of mycotoxins reduced eggshell strength by 12.3% (*P* < 0.05), and caused an accumulation of 249 μg/kg of DON in eggs at week 12 relative to the control group. Interestingly, dietary supplementation of TOXO HP mitigated DON-induced changes in eggshell strength, while dietary supplementation of either TOXO XL prevented accumulation of DON in the eggs (*P* < 0.05). For all treatments, AFB_1_ and OTA concentrations in eggs were lower than their respective detection limits of 2.0 and 1.9 μg/kg.

### 2.2. Organs Index and Serum Biochemistry

The relative organ weight results were presented in Table 3. Compared to the control, dietary supplementation of mycotoxins increased relative liver weights by 11.8% (*P* < 0.05), and decreased the spleen weights by 22.4% and relative proventriculus weights by 11.6% (*P* < 0.05). Interestingly, dietary supplementation with TOXO HP mitigated these mycotoxins-induced changes on all the organ indices, while supplementation with TOXO XL only prevented changes in liver and spleen indices (*P* < 0.05). Strikingly, compared to the control and toxin groups, the kidney and proventriculus indices were increased by 44.9–67.6% in Toxins + XL group (*P* < 0.05).

The serum biochemistry results are presented in Table 4. Compared to the control, dietary mycotoxin exposure increased the activity of ALT by 26.1% in serum (*P* < 0.05). Notably, dietary supplementation with mycotoxin binders prevented mycotoxin-induced alterations in serum ALT (*P* < 0.05). In contrast, dietary mycotoxins reduced serum concentrations of IgA, IgM, and IgG by 9.2–19.0% (*P* < 0.05). Generally, dietary supplementation with TOXO HP mitigated these mycotoxins-induced negative effects (*P* < 0.05). However, dietary supplementation with TOXO XL did not prevent the adverse effects of mycotoxins on antibody titers (*P* > 0.05).

### 2.3. Small Intestinal Morphology

**Figure 1:**
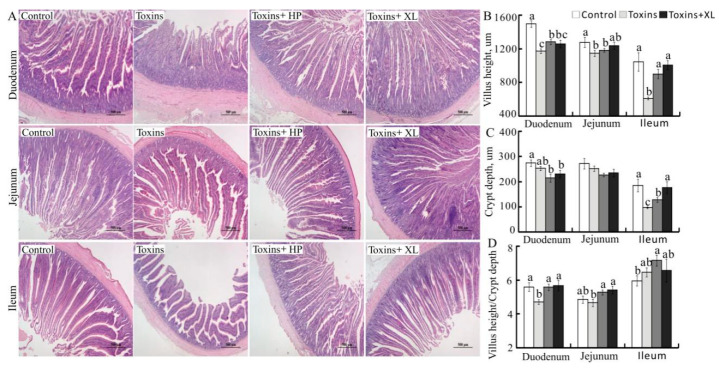
Effects of dietary mycotoxins and binders on histopathology of the duodenum, jejunum, and ileum (**A**) and morphology of the duodenum (**B**), jejunum (**C**), and ileum (**D**) of laying hens. Values are means ± SE, *n* = 10. Labeled means in a row without a common letter differ, *P* < 0.05. Control, basal diet; Toxins, basal diet supplemented with 0.15 mg/kg AFB_1_ + 1.5 mg/kg DON + 0.12 mg/kg OTA; Toxins + HP, basal diet supplemented with 0.15 mg/kg AFB_1_ + 1.5 mg/kg DON + 0.12 mg/kg OTA plus 0.29% TOXO HP; Toxins + XL, basal diet supplemented with 0.15 mg/kg AFB_1_ + 1.5 mg/kg DON + 0.12 mg/kg OTA plus 0.1% TOXO XL.

The small intestinal histopathology results are presented in Figure 1A. Compared to the control, dietary mycotoxins induced atrophy and fracture of intestinal villi, severe degeneration, necrosis, and desquamation of the villous epithelial cells, hemorrhage and inflammatory cell infiltration in submucosa and lamina propria, and (or) goblet cell hyperplasia in the intestinal gland in the duodenum, jejunum, and ileum. Generally, TOXO HP and XL mitigated most of the histopathological alterations induced by mycotoxins. The intestinal morphology results are presented in Figure 1B–D. Compared to the control, dietary supplementation of mycotoxins reduced villus height in duodenum, jejunum, and ileum, villus height/crypt depth in duodenum, and crypt depth in ileum (*P* < 0.05). Interestingly, TOXO HP and XL alleviated most of these adverse effects induced by mycotoxins in duodenum, jejunum, and (or) ileum (*P* < 0.05).

## 3. Discussion

The present study demonstrated that TOXO HP and XL binders could effectively counteract AFB_1_, DON, and OTA-induced adverse effects on laying hens. In agreement with previous studies [21,22,23], chicks exposed to AFB_1_, DON, and OTA, alone or in combination, reduced feed intake, total egg weight, and egg-laying rate, while they increased feed/egg ratio and mortality during various experimental periods. Meanwhile, mycotoxins also decreased the eggshell strength and increased DON residue in eggs relative to the control group, which was in agreement with previous reports [21,24,25,26]. The lower eggshell strength induced by mycotoxins was related to the impairment of calcium, zinc, vitamin A, E, and D3 metabolism [25]. However, the current study showed that internal egg quality traits, including albumen height, egg yolk color, and Haugh unit, were not affected by mycotoxins throughout the experiment duration, data were inconsistent with previous studies [25,27,28]. This divergence might be due to the differences in the domestic animal species, age, duration, and doses of mycotoxins. Generally, dietary supplementation of TOXO HP and XL binders successfully reduced most of these adverse effects induced by mycotoxins in laying hens. These outcomes were consistent with previous studies, which reported that bentonite or yeast products can alleviate mycotoxins-induced adverse effects on performance of livestock and poultry [19,29,30,31,32].

The poor performance of laying hens, induced by mycotoxins, was further proven to be associated with impairment of several organs in the present study. Specifically, compared to the control, dietary supplementation of mycotoxins increased relative liver weight, and reduced spleen and proventriculus relative weights. Moreover, serum biochemistry results showed that mycotoxins increased ALT activity while they decreased IgA, IgM, and IgG concentrations, which further confirmed the injury of liver and spleen of the laying hens [9,22,23]. These outcomes were similar to previous studies that showed livestock and poultry fed diets contaminated with AFB_1_, DON, and OTA, alone or in combination, displayed injury of liver, spleen, and stomach [8,9,11,12,33,34,35]. Interestingly, dietary supplementation of TOXO HP binder mitigated the mycotoxins-induced the negative changes on all the organs index and serum biochemistry. While, dietary supplementation of TOXO XL binder only mitigated the liver and spleen indexes and ALT activity changes induced by mycotoxins. These findings were similar to previous reports, which showed that bentonite, yeast cell wall and (or) β-1, 3/1, 6-glucans can mitigate mycotoxins-induced negative effects on various tissues and biochemical parameters of animals [19,29,30,31]. Notably, dietary supplementation of TOXO XL binder increased the relative weights of kidney and proventriculus than those of the control. This may be because the TOXO XL binder might cause side effects of swelling on kidney and proventriculus thus the attention should be paid [36,37].

The small intestine, including the duodenum, jejunum, and ileum, is the primary part where most nutrient digestion and absorption take place [8,38]. In the current study, the histopathological results showed that mycotoxins induced severe damage in small intestine of laying hens, as evidenced by villous atrophy and fracture, degeneration, necrosis, and desquamation of the villous epithelial cells, hemorrhage and inflammatory cell infiltration in submucosa and lamina propria, and (or) goblet cell hyperplasia in the intestinal gland, along with reduced villus height, villus height/crypt depth and crypt depth in the duodenum, jejunum, and (or) ileum. These findings were similar to previous studies that animals consumed diets contaminated with AFB_1_, DON, and OTA, alone or in combination, could induce intestine injury, thus reducing nutrient utilization efficiency and causing poor performance [23,39,40,41]. Interestingly, dietary supplementation with TOXO HP and XL binders alleviated mycotoxins-induced negative effects on histopathological alteration in small intestine of laying hens. These outcomes were in agreement with previous studies, which showed that dietary supplementation of yeast cell wall can improve the intestinal health and bentonite can reduce mycotoxins-induced adverse effects on intestine of chicks and pigs [19,30].

## 4. Conclusions

In summary, laying hens fed the diet co-contaminated with AFB_1_, DON, and OTA exhibited poor performance and egg quality and higher mortality. Furthermore, such adverse effects seem to be associated with the impairment of liver, spleen, proventriculus, and small intestine. Additionally, the current study has found that dietary supplementation with TOXO HP and XL binders could effectively counteract the combination of AFB_1_, DON, and OTA-induced negative effects on laying hens. Notably, XL was a better product in counteracting the negative effects of mycotoxins on performance parameters, while HP was a better product for immune modulation. Overall, these data indicated that the TOXO HP and XL binders could be used as promising binders for the control of mycotoxins in practice.

## 5. Materials and Methods

### 5.1. Birds, Treatment, Performance, and Sample Collection

The Institutional Animal Care and Use Committee of Huazhong Agricultural University, China supervised and approved the experimental protocol of this study. In total, 360 26-week-old Roman laying hens were randomly allocated into four experimental groups with 10 replicates of nine birds each. The four groups received either a basal diet (BD, Table 5; Control), BD supplemented with 0.15 mg/kg AFB_1_ + 1.5 mg/kg DON + 0.12 mg/kg OTA (Toxins), BD + Toxins with 0.29% TOXO HP (Trouw Nutrition, Amersfoort, The Netherlands; Toxins + HP), or a BD + Toxins with 0.1% TOXO XL (Trouw Nutrition, Amersfoort, The Netherlands; Toxins + XL). The doses were chosen on the basis of previous reports that showed that dietary consumption of 0.05–0.6 mg/kg AFB_1_, 0.35–4.5 mg/kg DON, or 0.16–0.33 mg/kg OTA alone can induce negative effects in laying hens [42,43,44,45,46]. The AFB_1_, DON and OTA were produced by *Aspergillus flavus* NRRL-3357, *Fusarium graminearum* W3008, and *Aspergillus ochraceus* AS3.3876, respectively, and mixed into the feeds, as described in previous studies [41,47,48]. The *Aspergillus flavus* NRRL-3357 and *Fusarium graminearum* W3008 were previously kept in our lab. The *Aspergillus ochraceus* AS3.3876 was generously donated by Dr. Wence Wang, College of Animal Science of South China Agricultural University. All birds were allowed access to the mash diets and water ad libitum for the 12-week exposure period. Feed intake, egg weight, and bird mortalities were recorded daily [49]. The feed/egg ratio, egg production rate, and interior egg quality were calculated every four weeks. Eggshell quality and egg mycotoxin concentrations were measured on the last day of the twelfth week [49]. At the end of the feeding trial, 10 birds from each group were slaughtered to collect blood samples, duodenum, jejunum, and ileum for serological and histological examination, as previously described [9].

### 5.2. Organ Index, Serum Biochemical, Mycotoxins, and Histological Analysis

The organ index was calculated according to the formula, organ index (g/kg) = organ weight/body weight [11,50]. The activities of aspartate aminotransferase (AST) and alanine aminotransferase (ALT), as well as concentrations of albumin (ALB) and total protein (TP) in serum were measured by an automatic biochemistry analyzer (Beckman Synchron CX4PRO, Brea, CA, USA). Total serumimmunoglobulin (Ig) A, IgM and IgG concentrations were measured with commercially available ELISA kits (A40199-S, A02721-S and A04022-S; Shanghai Jining Shiye Co. Ltd., Shanghai, China). Concentrations of AFB_1_, DON and OTA in whole egg were respectively measured with the commercially available ELISA kits COKAQ8000, COKAQ4000, and COKAQ2000 (Romer Labs Inc., Beijing, China). The detection limit of these ELISA kits for AFB_1_, DON and OTA are 2.0, 200 and 1.9 μg/kg, respectively. The small intestine tissues were examined microscopically after being fixed in 10% neutral buffered formalin, embedded in paraffin, sectioned at 5 μm, and then stained with hematoxylin and eosin [11].

### 5.3. Statistical Analysis

Statistical analysis of were performed using SPSS, version 13 (IBM, Chicago, IL, USA). Data were presented as mean ± SE. The Tukey-Kramer method was used for multiple mean comparisons. Significance was set at *P* < 0.10 for performance results and *P* < 0.05 for the rest of the results.

## Figures and Tables

**Table 1:** Effects of dietary mycotoxins and binders on performance of laying hens ^1^.

Index	Control	Toxins	Toxins + HP	Toxins + XL
Weeks 1–4				
Total feed intake, kg/hen	3.19 ± 0.05	3.16 ± 0.04	3.20 ± 0.03	3.21 ± 0.02
Total egg weight, kg/hen	1.44 ± 0.03	1.36 ± 0.04	1.41 ± 0.03	1.43 ± 0.04
Feed/egg ratio, kg/kg	2.23 ± 0.04	2.34 ± 0.06	2.28 ± 0.05	2.25 ± 0.05
Egg-laying rate, %	90.3 ± 1.5 ^a^	85.6 ± 2.0 ^b^	88.3 ± 2.0 ^ab^	89.2 ± 1.9 ^ab^
Mortality, %	0.00 ± 0.00 ^b^	3.33 ± 1.70 ^a^	0.00 ± 0.00 ^b^	2.22 ± 1.49 ^ab^
Weeks 5–8				
Total feed intake, kg/hen	3.21 ± 0.03 ^b^	3.26 ± 0.03 ^ab^	3.27 ± 0.01 ^a^	3.18 ± 0.04 ^b^
Total egg weight, kg/hen	1.50 ± 0.03	1.44 ± 0.02	1.46 ± 0.03	1.48 ± 0.02
Feed/egg ratio, kg/kg	2.14 ± 0.04 ^b^	2.27 ± 0.03 ^a^	2.24 ± 0.04 ^ab^	2.15 ± 0.03 ^b^
Egg-laying rate, %	91.8 ± 1.4 ^a^	86.7 ± 1.2 ^b^	89.2 ± 1.7 ^ab^	89.4 ± 1.0 ^a^
Mortality, %	1.23 ± 1.17	3.33 ± 1.70	3.33 ± 1.70	4.44 ± 2.46
Weeks 9–12				
Total feed intake, kg/hen	3.23 ± 0.02 ^a^	3.15 ± 0.02 ^b^	3.20 ± 0.03 ^ab^	3.16 ± 0.02 ^b^
Total egg weight, kg/hen	1.50 ± 0.04	1.43 ± 0.02	1.47 ± 0.03	1.48 ± 0.03
Feed/egg ratio, kg/kg	2.17 ± 0.05	2.21 ± 0.04	2.19 ± 0.04	2.15 ± 0.05
Egg-laying rate, %	89.9 ± 2.0 ^a^	85.2 ± 1.3 ^b^	86.9 ± 1.7 ^ab^	87.7 ± 1.5 ^ab^
Mortality, %	0.00 ± 0.00	1.11 ± 1.11	1.11 ± 1.11	0.00 ± 0.00
Weeks 1–12				
Total feed intake, kg/hen	9.64 ± 0.09	9.58 ± 0.09	9.67 ± 0.04	9.55 ± 0.09
Total egg weight, kg/hen	4.44 ± 0.09 ^a^	4.23 ± 0.05 ^b^	4.34 ± 0.07 ^ab^	4.39 ± 0.08 ^a^
Feed/egg ratio, kg/kg	2.18 ± 0.04 ^a^	2.26 ± 0.03 ^b^	2.23 ± 0.03 ^ab^	2.18 ± 0.04 ^a^
Egg-laying rate, %	90.6 ± 1.5 ^a^	85.8 ± 0.9 ^b^	88.1 ± 1.6 ^ab^	88.8 ± 1.1 ^a^
Mortality, %	1.23 ± 1.17 ^b^	7.78 ± 1.89 ^a^	4.44 ± 1.81 ^ab^	6.67 ± 2.96 ^ab^

^1^ Values are means ± SE, *n* = 10. Labeled means in a row without a common letter differ, *P* < 0.10. Control, basal diet; Toxins, basal diet supplemented with 0.15 mg/kg AFB_1_ + 1.5 mg/kg DON + 0.12 mg/kg OTA; Toxins + HP, basal diet supplemented with 0.15 mg/kg AFB_1_ + 1.5 mg/kg DON + 0.12 mg/kg OTA plus 0.29% TOXO HP; Toxins + XL, basal diet supplemented with 0.15 mg/kg AFB_1_ + 1.5 mg/kg DON + 0.12 mg/kg OTA plus 0.1% TOXO XL.

**Table 2:** Effects of dietary mycotoxins and binders on egg quality of laying hens ^1^.

Index	Control	Toxins	Toxins + HP	Toxins + XL
Week 4				
Egg weight, g	56.8 ± 0.6	56.8 ± 0.5	57.2 ± 0.5	57.2 ± 0.4
Albumen height, mm	8.00 ± 0.19	8.15 ± 0.14	8.19 ± 0.13	8.04 ± 0.12
Egg yolk color	8.26 ± 0.15	8.23 ± 0.07	8.25 ± 0.06	8.18 ± 0.06
Haugh unit	89.8 ± 1.1	90.8 ± 0.7	90.8 ± 0.6	90.1 ± 0.7
Week 8				
Egg weight, g	58.5 ± 0.6	59.4 ± 0.3	58.6 ± 0.4	59.3 ± 0.5
Albumen height, mm	7.13 ± 0.15	7.02 ± 0.14	7.21 ± 0.19	7.16 ± 0.13
Egg yolk color	8.38 ± 0.10	8.29 ± 0.08	8.48 ± 0.10	8.40 ± 0.10
Haugh unit	83.3 ± 0.9	83.0 ± 0.9	84.8 ± 1.1	84.9 ± 0.9
Week 12				
Egg weight, g	59.5 ± 0.6	60.0 ± 0.3	60.3 ± 0.5	60.2 ± 0.4
Albumen height, mm	7.52 ± 0.13	7.38 ± 0.09	7.35 ± 0.14	7.35 ± 0.14
Egg yolk color	6.89 ± 0.10 ^ab^	6.73 ± 0.11 ^b^	6.96 ± 0.08 ^ab^	7.09 ± 0.08 ^a^
Haugh unit	86.4 ± 0.9	85.6 ± 0.5	86.9 ± 0.7	85.4 ± 0.9
Eggshell strength, N	39.8 ± 0.8 ^a^	34.9 ± 1.5 ^b^	37.9 ± 1.0 ^ab^	35.3 ± 1.1 ^b^
DON residue in egg, μg/kg	– ^c,2^	249 ± 58 ^a^	228 ± 75 ^ab^	81 ± 36 ^b^

^1^ Values are means ± SE, *n* = 10. Labeled means in a row without a common letter differ, *P* < 0.05. Control, basal diet; Toxins, basal diet supplemented with 0.15 mg/kg AFB_1_ + 1.5 mg/kg DON + 0.12 mg/kg OTA; Toxins + HP, basal diet supplemented with 0.15 mg/kg AFB_1_ + 1.5 mg/kg DON + 0.12 mg/kg OTA plus 0.29% TOXO HP; Toxins + XL, basal diet supplemented with 0.15 mg/kg AFB_1_ + 1.5 mg/kg DON + 0.12 mg/kg OTA plus 0.1% TOXO XL. ^2^ “−” means lower than the detection limit of 200 μg/kg for DON.

**Table 3:** Effects of dietary mycotoxins and binders on relative organ weight of laying hens ^1.^

Index	Control	Toxins	Toxins + HP	Toxins + XL
Liver index, g/kg	19.5 ± 0.6 ^b^	21.8 ± 0.8 ^a^	19.6 ± 0.7 ^b^	18.9 ± 0.5 ^b^
Spleen index, g/kg	1.07 ± 0.05 ^a^	0.83 ± 0.05 ^b^	0.96 ± 0.06 ^a^	0.97 ± 0.05 ^a^
Kidney index, g/kg	3.07 ± 0.09 ^b^	3.23 ± 0.15 ^b^	3.45 ± 0.22 ^b^	4.68 ± 0.33 ^a^
Proventriculus index, g/kg	3.35 ± 0.15 ^b^	2.96 ± 0.09 ^c^	3.10 ± 0.09 ^bc^	4.96 ± 0.28 ^a^
Gizzard index, g/kg	11.2 ± 0.2	10.8 ± 0.6	10.9 ± 0.2	11.5 ± 0.5

^1^ Values are means ± SE, *n* = 10. Labeled means in a row without a common letter differ, *P* < 0.05. Control, basal diet; Toxins, basal diet supplemented with 0.15 mg/kg AFB_1_ + 1.5 mg/kg DON + 0.12 mg/kg OTA; Toxins + HP, basal diet supplemented with 0.15 mg/kg AFB_1_ + 1.5 mg/kg DON + 0.12 mg/kg OTA plus 0.29% TOXO HP; Toxins + XL, basal diet supplemented with 0.15 mg/kg AFB_1_ + 1.5 mg/kg DON + 0.12 mg/kg OTA plus 0.1% TOXO XL.

**Table 4:** Effects of dietary mycotoxins and binders on serum biochemistry of laying hens ^1.^

Index	Control	Toxins	Toxins + HP	Toxins + XL
ALT, U/L	2.1 ± 0.2 ^b^	2.9 ± 0.2 ^a^	2.3 ± 0.2 ^b^	1.8 ± 0.2 ^b^
AST, U/L	192.1 ± 6.2	203.6 ± 9.9	207.6 ± 7.1	207.2 ± 7.4
ALB, g/L	25.6 ± 0.5 ^b^	26.7 ± 0.5 ^ab^	25.9 ± 0.6 ^ab^	27.5 ± 0.6 ^a^
TP, g/L	52.3 ± 2.9	49.5 ± 3.7	49.2 ± 1.9	52.1 ± 4.4
IgA, μg/mL	343 ± 18 ^a^	278 ± 5 ^b^	352 ± 23 ^a^	279 ± 9 ^b^
IgM, μg/mL	764 ± 14 ^a^	694 ± 20 ^b^	769 ± 45 ^ab^	687 ± 13 ^b^
IgG, μg/mL	2831 ± 116 ^a^	2464 ± 63 ^b^	2810 ± 160 ^ab^	2244 ± 46 ^c^

^1^ Values are means ± SE, *n* = 10. Labeled means in a row without a common letter differ, *P* < 0.05. Control, basal diet; Toxins, basal diet supplemented with 0.15 mg/kg AFB_1_ + 1.5 mg/kg DON + 0.12 mg/kg OTA; Toxins+HP, basal diet supplemented with 0.15 mg/kg AFB_1_ + 1.5 mg/kg DON + 0.12 mg/kg OTA plus 0.29% TOXO HP; Toxins + XL, basal diet supplemented with 0.15 mg/kg AFB_1_ + 1.5 mg/kg DON + 0.12 mg/kg OTA plus 0.1% TOXO XL.

**Table 5:** Basal diet composition and nutrient level ^1^.

Ingredients	Proportion, %	Nutrients Level ^3^	Content
Corn	53.5	CP, %	15.2
Soybean meal 46%	23.5	ME, MJ·kg^−1^	14.59
Wheat bran	6.5	Ca, %	3.42
Soybean oil	5.0	TP, %	0.62
Salt	0.3	AP(P), %	0.39
Limestone	8.5	Lysine	0.91
DL-methionine	0.11	Methionine	0.42
Dicalcium phosphate	1.59		
Premix ^2^	1.0		
Total	100		

^1^ Control, basal diet; Toxins, basal diet supplemented with 0.15 mg/kg AFB_1_ + 1.5 mg/kg DON + 0.12 mg/kg OTA; Toxins + HP, basal diet supplemented with 0.15 mg/kg AFB_1_ + 1.5 mg/kg DON + 0.12 mg/kg OTA plus 0.29% TOXO HP; Toxins + XL, basal diet supplemented with 0.15 mg/kg AFB_1_ + 1.5 mg/kg DON + 0.12 mg/kg OTA plus 0.1% TOXO XL. The analyzed concentrations of AFB_1_, DON, and OTA were 0.003, 0.258, and 0.005 mg/kg in Control, 0.156, 1.784, and 0.122 mg/kg in Toxins, 0.150, 1.723, and 0.127 mg/kg in Toxins + HP, and 0.146, 1.790, and 0.120 mg/kg in Toxins + XL diets, respectively. ^2^ Provided the following per kg of diets: vitamin A, 12,000 IU; vitamin D_3_, 4000 IU; vitamin E, 35 IU; vitamin K, 5 mg; thiamine, 2 mg; riboflavin, 8 mg; vitamin B_6_, 5 mg; vitamin B_12_, 50 μg; D-biotin, 200 μg; pantothenic acid, 15 mg; nicotinic acid, 50 mg; choline, 500 mg; folic acid, 1.5 mg; Mn, (MnSO_4_, H2O), 120 mg; Zn (ZnO), 80 mg; Fe (FeSO_4_, H_2_O), 120 mg; Cu (CuSO_4_·5H_2_O), 15 mg; I (KI), 1 mg and Se (Na_2_SeO_3_), 0.3 mg. ^3^ Nutrient levels were a calculated value.

## Data Availability

The datasets used and/or analyzed during the current study are publicly available.
